# Long-term outcomes and clinical predictors of hospital mortality in very long stay intensive care unit patients: a cohort study

**DOI:** 10.1186/cc4888

**Published:** 2006-04-10

**Authors:** Jan O Friedrich, Gail Wilson, Clarence Chant

**Affiliations:** 1 Critical Care Department, St. Michael's Hospital, University of Toronto, Toronto, Canada; 2 Department of Medicine, St. Michael's Hospital, University of Toronto, Toronto, Canada; 3 Interdepartmental Division of Critical Care, University of Toronto, Toronto, Canada; 4 Leslie Dan Faculty of Pharmacy, University of Toronto, Toronto, Canada

## Abstract

**Introduction:**

Little information is available on prognosis and outcomes of very long stay intensive care unit (ICU) patients. The purpose of this study was to identify long-term outcomes after hospital discharge and readily available clinical predictors of hospital mortality for patients requiring prolonged care in the ICU.

**Method:**

Clinical data were collected from consecutive patients requiring at least 30 days of ICU care admitted over 3 calendar years (2001 to 2003) to a medical/surgical ICU in a university-affiliated tertiary care centre.

**Results:**

A total of 182 patients met the inclusion criteria, with a mean age of 63 years, median ICU stay of 48.5 days (interquartile range 36–78 days) and ICU mortality of 32%. They accounted for 8% of total admissions and 48% of total occupied beds. Of these patients, 42% died in hospital, 44% returned to their previous place of residence, and 14% were transferred to long-term care institutions. By 6 months after hospital discharge a further 8% of the patients had died, 40% remained at their previous place of residence, and 10% were in long-term care. Predictors of hospital mortality, identified using multivariate logistic regression, included age (odds ratio [OR] 1.45 per additional decade, 95% confidence interval [CI] 1.10–1.91), any immunosuppression (OR 5.2, 95% CI 1.7–15.5), mechanical ventilation for longer than 90 days (OR 4.0, 95% CI 1.3–12.0), treatment with inotropes or vasopressors for more than 3 days at or after day 30 in the ICU (OR 7.1, 95% CI 2.6–19.3), and acute renal failure requiring dialysis at or after day 30 in the ICU (OR 6.3, 95% CI 2.0–19.7).

**Conclusion:**

Patients with very long stays in the ICU appear to have a reasonable chance of survival, with most survivors in our cohort residing at their previous place of residence 6 months after hospital discharge. Prolonged requirement for life support therapies (ventilation, vasoactive agents, or acute dialysis) and a limited number of pre-existing co-morbidities (immunosuppression and, to a lesser extent, patient age) were predictors of increased hospital mortality. These predictors may assist in clinical decision making for this resource intensive patient population, and their reproducibility in other very long stay patient populations should be explored.

## Introduction

Long-stay intensive care unit (ICU) patients, variably defined as requiring longer than 5–14 days of intensive care, have been shown to have high mortality rates and consume significant resources [1-16]. Much less information is available on very long stay ICU patients, defined as requiring at least 28–30 days of ICU care [5,14,17-19]. The management of these patients can be particularly challenging for the multidisciplinary ICU team because of intense use of ICU resources that are limited, the challenges presented by a protracted weaning process, and uncertain long-term outcomes. Currently, prognostication for the very long stay ICU patient is imprecise. Most illness severity [20,21] or organ dysfunction [22,23] scoring systems were designed for patients with shorter ICU stays, and the predictive value of admission scoring systems based on acute physiological derangements decreases significantly beyond 7 days [24]. Given the challenges posed by this patient population and imprecise prognostication systems, the objectives of this study were as follows: to determine hospital and 6 month outcomes of a mixed population of medical/surgical patients requiring at least 30 days of ICU care; and to identify predictors associated with hospital mortality using ICU data readily available to clinicians at the bedside.

## Materials and methods

### Data sources

The study was conducted in the closed 24 bed medical/surgical ICU at St. Michael's Hospital, a tertiary-care academic centre affiliated to the University of Toronto. Patients requiring mechanical ventilation or intense physiological support or monitoring were admitted to the ICU and cared for by a multidisciplinary health care team under the direction of an attending intensive care physician. All decisions regarding patient care were made independent of data collection. The institution has separate neurosurgical/trauma, cardiac surgery and coronary care units that also accepted ventilated patients. Patients in these other units were only included in the study if they required transfer to the medical/surgical ICU for at least part of their hospital stay. No long-term ventilator unit exists within the institution, and so all ventilated patients remained in one of the acute care ICUs until transfer to a facility that could accommodate such patients.

### Data collection

Over a 3 year period (1 January 2001 to 31 December 2003), all admissions to the medical/surgical ICU were identified and all patients requiring at least 30 (consecutive or nonconsecutive) days of ICU care during their hospital admission were included in the study. Two study investigators (JF and GW) retrospectively reviewed each patient chart independently; disagreements were resolved by consensus. Baseline demographic data, including age, sex, body mass index, initial ICU admission diagnosis and severity of illness score (Acute Physiology and Chronic Health Evaluation [APACHE] II score [20]), were recorded at the time of initial ICU admission.

In addition, the presence of any baseline or ICU-acquired co-morbidities were recorded and grouped by system. The co-morbidities were selected *a priori *after informal discussion with intensive care physicians at the study hospital, who were asked to identify co-morbidities that contribute to increased length of stay or higher mortality in the very long stay patient population. These co-morbidities (with definitions) included the following: obesity (body mass index = 30 kg/m^2^), diabetes (by history, or if admitted with diabetic ketoacidosis or hypovolaemic hyperosmotic nonketotic coma, or if discharged on glucose lowering medications), chronic obstructive or restrictive lung disease (by history or radiographic imaging or pulmonary function testing), congestive heart failure (significant systolic or diastolic dysfunction by echocardiography), disabling neurological conditions (impaired cognition or muscle strength sufficient to impede ventilator weaning, as determined by review of the clinical notes), end-stage renal disease (requiring dialysis before admission), chronic liver disease (based on signs and symptoms of portal hypertension), any malignancy (excluding previously resected nonmelanoma skin cancers) and immunosuppression (ongoing requirement of any dose of steroids or other immunosuppressant medications, or HIV infection).

For each patient, we also recorded details of the ICU course (total number of ICU days, and days requiring invasive or noninvasive ventilatory support, renal replacement therapy for acute renal failure, and haemodynamic support with inotropes or vasopressors at any dose) and outcomes (ICU, hospital and 6 month mortality, and place of residence at hospital discharge and 6 months after discharge). Data regarding total length of ICU stay and days requiring life support therapies were incomplete for nine (5%) patients as a result of transfers between referring hospitals. However, demographics, co-morbidities, survival and place of residence up to 6 months after hospital discharge were available for all patients. For patients requiring more than one admission to the ICU during their hospital stay, their ICU course was recorded cumulatively, including data from all ICU admissions during the hospital stay.

The study protocol was approved by the hospital's research ethics board, which waived the need for informed consent.

## Statistical analyses

Agreement for recorded data between two investigators was evaluated using the kappa statistic for categorical variables, and Pearson correlation and paired *t *test for APACHE II scores. Baseline demographics, co-morbidities, and durations of ICU stay and life support therapies for hospital survivors and nonsurvivors were analyzed using Student's *t *or Wilcoxon tests for normally and non-normally distributed continuous variables, respectively. χ^2 ^or Fisher's exact test were used for categorical variables. Continuous variables are summarized as mean ± standard deviation or median (interquartile range [IQR]) for normally and non-normally distributed variables, respectively.

For multivariate analysis, the skewed continuous variables (ICU readmissions and days requiring ICU care, mechanical ventilation, inotropes or vasopressors, or dialysis) were converted to binary variables that would be easily available to bedside clinicians for patients requiring at least 30 days of ICU care; the approach was as follows. Cutoff points for number of ICU readmissions, ICU length of stay and duration of ventilation (both in multiples of 30 days) were chosen as the values after which there was the greatest change in hospital survival. We defined *a priori *haemodynamic support as the requirement of inotropic or vasopressor agents on at least 3 (consecutive or nonconsecutive) days from day 30 in ICU onward (to exclude patients requiring only a very brief period of haemodynamic support after day 30). Similarly, acute renal failure was defined as the requirement for dialysis in the ICU after day 30.

All variables with a *P *value below 0.20 by univariate logistic regression analysis were entered into a multivariate logistic regression model using backward selection. Variables with a *P *value below 0.10 were retained in the multivariate model, and these retained variables were assessed for collinearity or the presence of significant second-order interactions. Discrimination of the model was assessed by the area under the receiver operating characteristic curve. Calibration was assessed using the Hosmer and Lemeshow X^2 ^statistic. We report odds ratio (OR) and 95% confidence interval (CI), and interpreted two-sided *P *< 0.05 as being statistically significant. All statistical calculations were carried out using SAS version 8.2 (SAS Institute Inc., Cary, NC, USA).

## Results

Over the 3-year study period there were a total of 3,172 admissions; of these, 2,716 patients survived (ICU mortality rate 14%). There were 171 patients who had at least one stay of 30 or more consecutive days in ICU, and 11 patients who had more than one admission adding up to 30 or more total days in ICU. These 182 patients had a total of 266 ICU admissions during their hospital stay (median total ICU stay 48.5 days, IQR 36–78 days; and median total hospital stay 85 days, IQR 56–133 days). ICU mortality in these patients was 32%. This cohort represented 8% of total admissions but occupied 48% of the total bed days.

Patient demographics, co-morbidites and characteristics at ICU admission of the hospital survivors and nonsurvivors are shown in Table 1. There was good agreement between raters for each of the co-morbidity characteristics (kappa ranged from 0.75 to 1.00) and admission APACHE II scores (correlation coefficient *r *= 0.87), with an average difference in scores of 2.1 ± 4.3 (*P *= 0.002). Most (>90%) of the very long stay patients lived independently before their ICU admission. The ICU admissions were emergent in the majority of cases with a mean APACHE II score of 24 ± 8. The median number of co-morbidities was 2 (IQR 1–2).

These very long stay patients had the following outcomes: 42% died in hospital, 44% were discharged either to their previous place of residence or rehabilitation (with the expectation that they would return to their previous place of residence), and the remaining 14% were discharged to long-term care institutions with the expectation that these patients would require care in these long-term care institutions indefinitely. Of the 14% discharged to long-term care institutions, 6% were discharged with no permanent tracheostomy, 6% were discharged with a permanent tracheostomy but not chronically ventilated, and 2% were chronically ventilated. Fourteen patients (8% of the entire cohort) died during the 6 months following hospital discharge and one patient successfully returned home from a long-term care institution. Thus, at 6 months, 50% of patients had died, 40% were living in their previous place of residence, and 10% were living in long-term care institutions.

Details regarding patients' ICU course are shown in Table 2. All but one patient required ventilation; patients had a median of 7 (IQR 3-13.5) nonventilated days in ICU. Seventy per cent of patients required inotrope or vasopressor support at some point while they were in the ICU, and 16% required dialysis for acute renal failure. Most patients who required inotrope or vasopressor support early during their ICU admission no longer required such support from day 30 onward. In contrast, most patients dialyzed for acute renal failure still required this therapy at or after day 30. However, all survivors to 6 months after hospital discharge who had required dialysis for acute renal failure recovered renal function.

**Figure 1:**
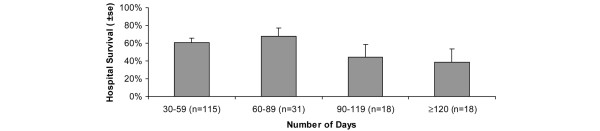
Hospital survival by number of ICU days. ICU, intensive care unit; se, standard error.

**Figure 2:**
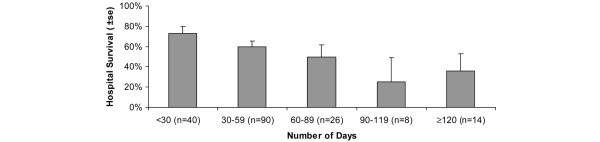
Hospital survival by number of ventilator days. se, standard error.

**Figure 3:**
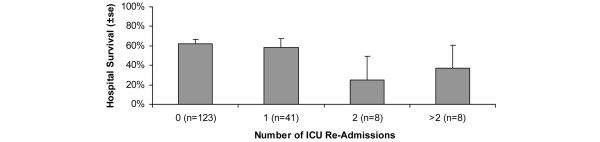
Hospital survival by number of ICU readmissions during the hospital stay. ICU, intensive care unit; se, standard error.

Compared with those who survived to hospital discharge, nonsurvivors were older, and more of these patients had diabetes, congestive heart failure, or were immunocompromised (Table 1). The nonsurvivors also had higher APACHE II scores at ICU admission, more readmissions, more ICU and ventilator days, and more of these patients required inotrope or vasopressor support or dialysis (Tables 1 and 2). The most significant change in hospital survival occurred after 90 days in the ICU (Figure 1; *P *= 0.02) or 90 days of ventilation (Figure 2; *P *= 0.007). Hospital survival was not affected by one ICU readmission, but multiple ICU readmissions were associated with increasing risk for mortality (Figure 3; *P *= 0.02).

**Figure 4:**
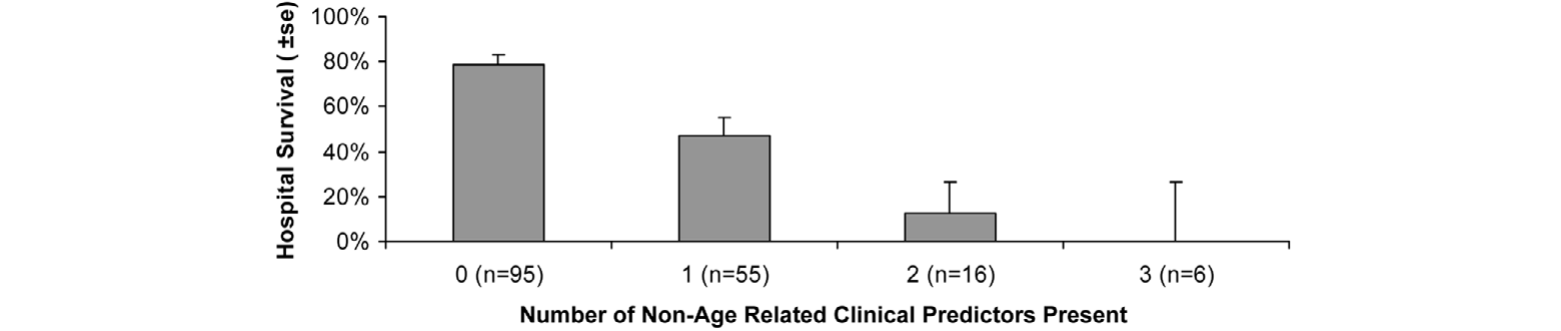
Hospital survival by number of non-age-related clinical predictors identified by multivariate logistic regression analysis. ICU, intensive care unit; se, standard error.

Based on these results, the following variables were entered into the multivariate logistic regression model: the continuous variables of age and APACHE II score; and the binary variables of diabetes, congestive heart failure, immunosuppression, more than one readmission to ICU, ICU length of stay greater than 90 days, duration of ventilation greater than 90 days, requirement for inotropic support on at least 3 days from day 30 in the ICU, and acute renal failure requiring dialysis in ICU from day 30. The results of the multivariate logistic regression model identifying predictors of hospital survival are shown in Table 3. Increasing age (OR 1.45 per incremental decade, 95% CI 1.10–1.91), immunosuppression (OR 5.2, 95% CI 1.7–15.5), more than 90 ventilator days (OR 4.0, 95% CI 1.3–12.0), acute renal failure requiring dialysis at or after day 30 (OR 6.3, 95% CI 2.0–19.7), and inotropic support on at least 3 days at or after day 30 in ICU (OR 7.1, 95% CI 2.6–19.3) were all independent predictors of higher hospital mortality. The area under the receiver operating characteristic curve for this model was 0.80. There was no evidence of lack of calibration (*P *= 0.29 for the final model), and there was no significant collinearity or second-order interactions among these variables. Hospital survival decreased markedly as the number of non-age-related clinical predictors of hospital mortality increased, from 75 out of 95 (79%) with no predictors to 26 out of 55 (47%) with one predictor, two out of 16 (13%) with two predictors, and none out of six (0%) with three predictors (Figure 4).

## Discussion

The major findings of this study are as follows. Patients who required at least 30 days of ICU care during their hospital stay comprised only a small proportion of total ICU admissions but they occupied a large proportion of total bed days. More than half of these very long stay patients survived to hospital discharge, with the vast majority returning to their previous place of residence by 6 months after hospital discharge. Independent predictors of hospital mortality in these patients were age, immunosuppression, greater than 90 ventilator days, acute renal failure requiring dialysis at or after day 30 in the ICU, and inotrope support on at least 3 days at or after day 30 in the ICU.

The finding that very long stay patients consumed a high proportion of total patient days is consistent with those of other studies. Studies conducted in medical/surgical, cardiovascular and pediatric ICU patients using shorter (7–14 days) definitions of long stay found that these patients accounted for only 4–11% of admissions while consuming 28–53% of available bed days [5,8,9,15,25-28]. Moreover, our observed hospital mortality (42%) is comparable with rates reported in other studies (40–53%) enrolling patients with at least 28–30 days in ICU [5,17-19]. Longer term (6 month to 1 year) mortality rates of 55–57% in recent studies enrolling patients after 14 days in ICU or 14 days of ventilation [7,13,16] were similar to our finding of 50% mortality at 6 months after hospital discharge. Six month outcomes from our study may be more similar to longer term outcomes in hospital systems commonly used in the USA, where patients are discharged from an acute care hospital to a long-term acute care or weaning centre earlier than in our study.

Five factors were found to be independently associated with hospital mortality in very long stay patients. The information required to determine whether each of these factors is applicable to a particular patient is readily available to clinicians. Hospital survival decreased markedly as the number of non-age-related hospital mortality predictors increased. However, cautious extrapolation of these findings to other patients is warranted, given the relatively small number of patients with more than one non-age-related mortality predictor in the study cohort.

It may be difficult to discuss changing the focus of care with patients and their families after 30 days in ICU when extensive resources have already been invested. However, given the prognostic implications, if the introduction of new life support therapies in this patient population becomes necessary because of clinical deterioration, then the outcome information derived from this study can help to further inform and guide the decision-making process.

Although the effect of age is a statistically significant factor, its effect is considerably smaller than that of the other factors. For example, a patient 20 years older than the average age in our cohort of 63 who survived to 30 days in ICU would still have an expected hospital survival of about 40%, only somewhat lower than the overall cohort average of 58% (i.e. an OR of mortality of [1.45]^2 ^for two decades). Furthermore, other than immunosuppression, no other co-morbidities were consistently found to have a large impact on survival. A potential explanation for this finding is that baseline co-morbidities are important predictors of short-term survival, but patients surviving to 30 days have demonstrated sufficient physiological reserve, despite their advanced age or any other co-morbidities. New physiological derangements, as reflected in ongoing or new requirements for life support therapies, become the important factors associated with survival.

A few studies, primarily in cardiac surgery or trauma patients and in long-term ventilator weaning centres, have identified similar predictors of poor outcome including age [5,12,16,19,28-30], inotrope requirements or nosocomial sepsis [5,16], immunosuppression [16], need for renal replacement therapy [10,16], and multiple organ failures [31]. One study [17] did not find total days of mechanical ventilation to be related to survival; however, that study only compared patients ventilated for 29–46 days versus those ventilated 47 or more days, and did not further separate out patients ventilated for longer time periods.

The present study has a number of limitations. The data generated may not be generalizable because they are derived from the experience of a single centre and reflect a unique organization and process of care. For example, the results may not directly apply to hospital systems with intermediate care/step down units for high intensity patients not requiring mechanical ventilation, or for hospital systems with more readily available chronic ventilator units. A factor that may affect outcomes is the approach to treatment limitation, which is likely to differ at least to some degree between centres. Although the practice in our centre is not to continue life support therapies in cases of futility, this is a complex issue influenced by patient and family values and with some variability in opinion and practice between individual clinicians. Furthermore, our hospital does not perform solid organ transplant surgery (other than kidneys) and the medical/surgical ICU does not routinely admit cardiac surgery or neurosurgical/trauma patients, and so the results may not be applicable to these patient groups. However, other studies identifying predictors of outcome in cardiac surgery [5], some including heart and lung transplantation [16], and trauma patients [12] have identified similar prognostic factors. This may suggest that once patients have overcome their initial reason for ICU admission (complex surgery, trauma, sepsis and so on.) and have survived to 30 days, they begin to face similar issues associated with prolonged dependence on life support (for example secondary infections and complications secondary to prolonged immobility).

Although the number of patients enrolled in the present study is relatively large, given the rarity of patients with very long ICU stays, it is still a relatively small number from a statistical perspective, which limits the precision of the OR estimates for the predictors and reduces the power to detect predictors with lower ORs. Furthermore, it is difficult to correlate the severity of the risk factor to outcome (for example the degree of immunosuppression) or to determine whether subcategories are important (such as lung cancer compared with other types of cancer) [32]. A further loss of precision may have occurred by changing continuous predictor variables into binary variables (which were chosen to be more readily available to clinicians at the bedside). Other factors, some of which are difficult to measure objectively, that may influence patient outcomes were not recorded in our data collection. These include the use of neuromuscular blocking agents, which were infrequently used in our patients, presence of delirium and high sedative use.

Finally, quality of life in survivors was not measured. Many previous studies have shown that quality of life in patients surviving long ICU stays is reasonably good [7,11,26,33-37]. Although quality of life was not measured in our study, most survivors returned to their previous place of residence, which is an important quality of life indicator for many critically ill patients and their families.

## Conclusion

In summary, the outcome predictors identified in this study are readily available to treating physicians and suggest that prolonged requirements for various life support therapies (ventilation, inotropes or vasopressors, and acute dialysis), in addition to immunosuppression and (to a lesser extent) patient age, are more important than other pre-existing co-morbidities for determining hospital mortality in very long stay ICU patients. Clinical decisions regarding individual very long stay patients clearly require incorporating other individualized clinical information in addition to patient values and beliefs. However, the predictors identified in this study may assist this decision making process, and their reproducibility in other very long stay patient populations should be explored. The identification of accurate predictors of hospital survival is important because the decision to withdraw or limit life-sustaining therapy in ICU patients is greatly influenced by physician's prediction of patients' ability to survive their ICU stay [38,39]. It is particularly important for this patient population because outcomes are favourable in many cases, with around half of the patients surviving and the vast majority of survivors returning to their previous place of residence and remaining there for at least 6 months after hospital discharge.

## Key messages

• 	ICU patients admitted over a 3-year period with very long lengths of stay (= 30 days in the ICU) accounted for only 8% of total admissions but 48% of total occupied beds.

• 	Six months after hospital discharge, 50% of the cohort was still alive and 80% of the survivors were living in their previous place of residence.

• 	Prolonged requirements for life support therapies (mechanical ventilation, inotropes/vasopressors, or acute dialysis) and a limited number of pre-existing co-morbidities (immunosuppression and, to a lesser extent, patient age) were predictors of increased hospital mortality.

• 	These predictors may assist in clinical decision making for this resource intensive patient population, and their reproducibility in other very long stay patient populations should be explored.

## Abbreviations

APACHE = Acute Physiology and Chronic Health Evaluation; CI = confidence interval; ICU = intensive care unit; IQR = interquartile range; OR = odds ratio.

## Competing interests

The authors declare that they have no competing interests.

## Authors' contributions

JF was involved in the conception and design of the study, acquisition, analysis and interpretation of data, and wrote the first draft of the manuscript. GW was involved in the conception and design of the study, acquisition and interpretation of data, and critical revision of the manuscript for important intellectual content. CC was involved in the acquisition and interpretation of data, and critical revision of the manuscript for important intellectual content. All authors read and approved the final version of the manuscript.

## Figures and Tables

**Table 1:** Patient characteristics

Characteristic	Survivors (*n *= 106)	Nonsurvivors (*n *= 76)	*P*
Comorbidites			
Age (years [mean ± SD])	61 ± 17	65 ± 15	0.11
Female sex	40 (38%)	28 (37%)	0.90
Obese (i.e. BMI > 30 kg/m^2^)	27 (26%)	17 (22%)	0.58
Diabetes	16 (15%)	23 (30%)	0.014
Congestive heart failure	16 (15%)	19 (25%)	0.09
Disabling neurological disease	33 (31%)	22 (29%)	0.75
Lung disease	32 (30%)	25 (33%)	0.70
ESRD	4 (4%)	5 (7%)	0.39
Liver disease	2 (2%)	3 (4%)	0.65^a^
Immunocompromised	10 (9%)	16 (21%)	0.03
Cancer	15 (14%)	12 (16%)	0.76
ICU admission			
Place of residence before hospital admission			
Home	99 (93%)	70 (92%)	0.48^a^
Nursing home^b^	4 (4%)	5 (7%)	
Tracheostomy^c^	3 (3%)	0 (0%)	
Ventilated^d^	0 (0%)	1 (1%)	
Admission APACHE II score (mean ± SD)	23 ± 8	26 ± 9	0.005
Medical admission	58 (55%)	46 (61%)	0.43
Nonelective admission	88 (83%)	63 (83%)	0.98
Admission diagnostic group			0.21^a^
Respiratory	35 (33%)	31 (41%)	
Cardiovascular	24 (23%)	21 (28%)	
Gastrointestinal	29 (27%)	9 (12%)	
Neurological	11 (10%)	4 (5%)	
Genitourinary	2 (2%)	6 (8%)	
Other	5 (5%)	5 (7%)	

Values are expressed as *n *(%) except where noted otherwise. ^a^*P *value obtained using Fisher's exact test. ^b^'Nursing home' refers to a patient from a long-term care institution without a permanent tracheostomy. ^c^'Tracheostomy' refers to a nonventilated patient with a permanent tracheostomy from a long-term care institution. ^d^'Ventilated' refers to a chronically ventilated patient from a long-term care institution. APACHE, Acute Physiology and Chronic Health Evaluation; BMI, body mass index; COPD, chronic obstructive pulmonary disease; ESRD, end-stage renal disease; ICU, intensive care unit; SD, standard deviation.

**Table 2:** Intensive care unit course

Characteristic	Survivors (*n *= 106)	Nonsurvivors (*n *= 76)	*P*
Number of ICU readmissions			
0	76 (72%)	47 (63%)	0.07^a^
1	24 (23%)	17 (23%)	
2	2 (2%)	6 (8%)	
>2	3 (3%)	4 (6%)	
Total duration of ICU stay (days)			
Median	48	54	0.14^b^
Interquartile range	35–70	36–96	
Requiring ventilation	106 (100%)	75 (99%)	0.42^c^
Total duration of ventilation (days)			
Median	39	46	0.002^d^
Interquartile range	29–53	34–86	
Requiring inotropes/vasopressors at any time in the ICU	65 (61%)	64 (84%)	0.0008
Requiring inotropes/vasopressors for at least 3 days at or after day 30	8/103 (8%)	26/70 (37%)	<0.0001
ARF requiring dialysis at any time in ICU (excluding ESRD)	6 (6%)	23/75 (30%)	<0.0001
ARF requiring dialysis at or after day 30 (excluding ESRD)	5 (5%)	22/75 (29%)	<0.0001

Values are expressed as *n *(%) except where noted otherwise. ^a^*P *= 0.02 using χ^2 ^test comparing patients with 0–1 readmissions versus patients with >1 readmissions (also see Figure 3). ^b^*P *value obtained using Wilcoxon Rank Sum test; *P *= 0.02 using χ^2 ^test comparing patients requiring <90 days of ICU care versus patient requiring >90 days of ICU care (also see Figure 1). ^c^*P *value obtained using Fisher's exact test. ^d^*P *value obtained using Wilcoxon rank sum test; *P *= 0.007 using χ^2 ^test comparing patients requiring <90 days of ventilation versus patient requiring >90 days of ventilation (also see Figure 2). ARF, acute renal failure; ESRD, end-stage renal disease; ICU, intensive care unit.

**Table 3:** Results of multivariate logistic regression analysis of patient characteristics associated with hospital mortality

Clinical mortality predictor	OR (95% CI)	*P*
Age (per 10-year increase)	1.45 (1.10–1.91)	0.009
Immunocompromised	5.2 (1.7–15.5)	0.003
>90 Ventilator days	4.0 (1.3–12.0)	0.013
3 days of inotropes/vasopressors from day 30 in ICU	7.1 (2.6–19.3)	0.0001
ARF requiring dialysis from day 30 in ICU (excluding ESRD patients)	6.3 (2.0–19.7)	0.0015

ARF, acute renal failure; CI, confidence interval; ESRD, end-stage renal disease; ICU, intensive care unit; OR, odds ratio.
